# How violence against women and girls undermines resilience to climate risks in Chad

**DOI:** 10.1111/disa.12343

**Published:** 2019-04-04

**Authors:** Virginie Le Masson, Colette Benoudji, Sandra Sotelo Reyes, Giselle Bernard

**Affiliations:** ^1^ Research Associate at the Overseas Development Institute United Kingdom; ^2^ Coordinator, Lead Tchad Chad; ^3^ Gender Justice and Resilience Advisor at Oxfam Spain; ^4^ Postgraduate student at the University of Oxford United Kingdom

**Keywords:** Chad, gender equality, gender‐based violence, resilience, risks

## Abstract

What consequences does ‘everyday violence’ have on the abilities of survivors to protect themselves from further risks? This paper seeks to establish the linkages between violence and people's resilience capacities to survive and adapt to environmental changes, particularly those living in fragile economic and political contexts such as Chad. It investigates not only how the adverse consequences of violence against women and girls affect the health status and livelihoods of survivors, but also their capacities, and those of their household and community members, to further protect themselves from other risks. Empirical evidence collected in Chad as part of the BRACED (Building Resilience and Adaptation to Climate Extremes and Disasters) programme shows that ‘everyday violence’ undermines resilience‐building at the individual, household, and community level. These results have serious implications for development programmes and the role they need to play to better promote both gender equality and resilience to shocks and stresses.

## Introduction

Resilience‐building programmes designed to help people affected by disasters and climate change are increasingly targeting women. This emphasis on gender issues in resilience programmes draws on the literature documenting the widespread socioeconomic inequalities that disproportionately affect women and aggravate their vulnerabilities to disaster risks (Enarson and Morrow, 1998; Bradshaw and Fordham, 2013). Contextual analyses conducted by development actors also reveal how discrimination against women's perceived status in society further undermines their resilience capacities (Mercy Corps, 2014; Morchain et al., 2015; Opondo et al., 2015).

As a response, the integration of gender‐sensitive approaches in development projects aims to make inequalities more visible, reduce the vulnerabilities of the most marginalised members of communities and build people's resilience to shocks and stresses. Such approaches are also framed by human rights‐based principles that consider that people affected by crisis are not only recipients of aid but also holders of rights that must be respected and protected (Sotelo, 2017). To guarantee the exercise of these rights, processes of exclusion, power relations and resulting inequalities must therefore be acknowledged and tackled.

The recognition of gender considerations in resilience programming aims to address these inequalities but it does not systematically integrate issues of violence. Yet there is an extensive literature documenting the prevalence of gender‐based violence (GBV) and violence against women and girls (VAWG) globally (WHO, 2013; IASC, 2015); in conflict‐affected settings (Human Security Research Group, 2012; WhatWorks, 2017); and in the aftermath of disasters (see Enarson (2006) for a review; Amnesty International, 2011a; IFRC, 2016). Violence perpetrated against a person because of their gender is a sexist phenomenon, rooted in the inequality that exists between men and women around the world (Baker and Cunningham, 2005). The Istanbul Convention recognises that violence against women is one of the social mechanisms by means of which women have historically been held in a subordinate position to men (Council of Europe, 2011). Based on this principle, this paper uses the term VAWG, but it recognises that men and boys are also at risk of experiencing GBV (UN Women, 2015).

Overall, evidence on the occurrence of violence in emergencies is gathered mostly by agencies involved in relief work and in the protection sector, and it underscores that post‐disaster GBV occurs in all countries and at all stages of development (Amnesty International, 2011; Parkinson, 2015; IFRC, 2016). Fewer studies, however, examine the consequences of everyday violence – that is, violence that is not necessarily perpetrated in wartime and by combatants but by household and community members on a daily basis. There is even less research on the noticeable effects this violence may have on the physical and psychosocial health of survivors (Gibbs et al., 2018) and their ability to protect themselves from further risks. Yet such evidence is crucial to better establish the linkages between the consequences of violence and survivors’ access to livelihoods, and therefore their resilience capacities to survive and adapt to environmental changes, particularly if they live in fragile economic and political contexts (Koester et al., 2016).

Another issue lies in the fact that the social and gender aspects of vulnerability to climate change and disaster risks remain all too often simplified to targeting women and girls as one vulnerable group (Fordham, 1999). They become the target recipients of humanitarian aid and development projects that aim to increase their economic resources, but the factors that sustain the exclusion of people of a particular gender, age or ethnicity background, that expose them to insecurity and that prevent them from being fully involved in the decisions that affect their lives are rarely addressed. These factors include social norms that tolerate the domination of one gender above the other. Despite the scale of the problem, ‘everyday’ violence remains confined to the private and familial sphere, in which external actors do not necessarily want to get involved.

Recognising that the consequences of violence negatively affect the health status and livelihoods of survivors, this paper aims to examine how these adverse consequences further affect the resilience capacities not only of survivors of violence but also of their household and community members, to face external shocks and stresses. It brings in empirical evidence collected in Chad as part of the Building Resilience and Adaptation to Climate Extremes and Disasters (BRACED) programme, and investigates the extent to which ‘everyday violence’ undermines resilience‐building, at the individual, household and community levels. These results have serious implications for development programmes and the role they need to play to better promote both gender equality and resilience to shocks and stresses.

## Methodology

To examine the impact of VAWG on survivors’ abilities (and those of their household and their community) to protect themselves from disasters and adapt to environmental changes, this study documents the Chadian context and draws on the analysis of both secondary and primary data. Secondary data come from non‐governmental organisation (NGO) programming and reporting documents as well as the 2014–2015 Demographic and Health Survey, which synthesises the prevalence of violence against women in Chad (INSEED et al., 2015, referred to here as EDS/MICS 2014–2015).

Primary data were collected through qualitative methods and fieldwork carried out in Chad between March and June 2017. A total of 38 semi‐structured interviews were held with key informants, including national and international staff from NGOs and UN agencies working on resilience and protection, representatives of local and regional authorities and members of village associations. Villagers and survivors of violence were also interviewed in accordance with the recommendations established by the World Health Organization (WHO) in terms of ethics, participants’ safety and quality of information (WHO, 2007). Ten focus groups were held with women, men and youth groups to further explore *how* violence affects the resilience of survivors and those who live with them.

The study sites included the regions of Dar Dar Sila and Bahr‐el‐Gazal (BeG), and the capital, N'Djamena. The total number of participants was 162 people (115 women and 47 men), selected partly through targeted sampling informed by NGO staff members and local individuals in positions of influence. For community participants, ‘snowball’ sampling was used to approach those recommended by interview participants or NGOs. Quoting is anonymous, to protect the identity of participants, and accounts were recorded with a recording device and/or written down with participants’ consent.

Research findings on the prevalence of VAWG, the risk factors and the processes that seek to exclude women and girls in Chad are presented in detail in a BRACED working paper (Le Masson et al., 2018). The following analysis draws on these results but focuses on documenting the linkages between the impacts of violence on the livelihoods of survivors and the wider implications for their capacities and those of their households and communities to build their resilience.

The concept of resilience refers to the ability of individuals, their homes and their communities to anticipate, avoid, confront, recover from and adapt to natural hazards and environmental changes (DFID, 2014) so that impacts are the least destructive possible. The ability to cope with crises, whether sudden (such as flood damage) or prolonged (such as a famine that lasts years), stems from resilience capacities that individual and collective actions can improve. We base this conceptualisation on Oxfam's approach to resilience‐based development (Jeans et al., 2016), which views resilience through the development of three capacities.

**Absorptive capacity** refers to the ability to develop protective actions to cope with traumatic events and crises that may continue to occur as a result of climate change, prolonged conflicts or chronic food shortages. **Adaptive capacity** makes it possible to develop new strategies to better manage natural hazards and progressive adjustments in anticipation of, or in response to, a change, in order to create greater flexibility. **Transformative capacity** is the ability to affect economic, social, cultural or political change aimed at eliminating or reducing vulnerability, and hence the risk of being affected by a crisis. Transformation involves reducing inequalities because people who are socially, culturally, financially and politically marginalised are also more vulnerable to risk (Pelling, 2011). The transformation of unjust social relations or inequalities of power is thus necessary to build the resilience of everyone to survive a crisis.

## The context of violence against women and girls in Chad

The population in Chad faces a wide range of risks, whose effects are mutually reinforcing: recurring droughts; severe food shortages that affect one in five people (OCHA, 2016); politico‐military instability and subsequent population displacement (Favre, 2007; Médard and Ozias, 2007); rising food prices (following closure of the Chadian border with Libya in January 2017) (APA, 2017); extreme rainfall and floods (RFI, 2014); and other negative effects of climate change (Republic of Chad, 2010). Facing these risks, the country's vulnerability profile is high (World Bank, 2016). Between 47% and 55% of the Chadian population is considered poor, depending on the sources and indicators of poverty being used (OCHA, 2016; World Bank, 2016) and 4.7 million people needed humanitarian assistance in 2017 – that is, 32% of the country's total population (OCHA, 2016) (see Table 1 for an overview of socioeconomic characteristics).

**Table 1:** Socioeconomic characteristics in Chad and in the two regions of study

	National	Dar Dar Sila	BeG
Population in moderate to severe food insecurity (in 2014)	23%	19%	49%
Electricity access (% of households)	7.7% (49.6% in N'Djamena)	NA	NA
Households practising open defecation (%)	70.7% (3% in N'Djamena)	NA	NA
Female‐headed households (%)	22%	NA	NA
Literacy rate among 15–24 year olds	35.3% of women	NA	NA
	64.8 of men		
Percentage of people who never went to school	59.5% of women	82.2% of women	83.4% of women
	43.2% of men	64.6% of men	79% of men
Women who reported domestic violence (whether physical, sexual or psychological)	34.8%	22.4%	22.9%
Women who reported having experienced sexual violence at least once in their life	11.6%	11.1%	18%
Women who reported having experienced physical violence at least once in their life	29	21.3%	21%

**Sources**: authors, based on EDS/MICS 2014–2015; World Bank (2016).

In addition to conflict‐related violence occurring in Chad, there exists a considerable amount of non‐conflict‐related violence, mostly against women and girls. Nationally, more than a third (35%) of women aged 15–49 living in a relationship report having been victims of physical, psychological and/or sexual violence by their spouses at least once in their lifetime (EDS/MICS 2014–2015). Among Chadian women who reported having experienced domestic violence, more than half (51%) had been injured in the previous 12 months as a result of physical or sexual violence by their spouse (EDS/MICS 2014–2015).

Accounts from Dar Dar Sila and BeG echo the national statistics (although the latter are likely to be underestimated). Participants in a discussion in Dar Sila spoke of women who had had to be taken to the hospital and/or bedridden as a result of violent beatings by their husbands. The most common forms of violence reported were early (child) marriage, physical and sexual violence perpetrated by relatives, polygamy and the associated discrimination against women (such as the risk of becoming divorced and homeless) and the denial of resources and opportunities by women's husbands and relatives (see Table 2).

**Table 2:** Cases of violence against women and children, as reported in interviews

	Abuse of power and domination			
Controlling behaviour	Psychological violence	Economic violence	Physical violence	Sexual violence
Forcing girls and women to marry Preventing girls from attending school Preventing women from going to health centre Preventing an abortion or forcing women to abort Forcing women to stay at home Preventing women from accessing contraception Preventing women or their families from complaining to the authorities Preventing women from getting divorced Controlling the resources of the home Dominating decision‐making in the home Polygamy	Scorn, denigration Abandoning the home Threatening to marry another woman Reminding women they are inferior to men Insults Blaming a woman for bringing shame on family when she is raped Separating a girl from her parents when she is raped Harassing single women in the public space Divorce because a woman is HIV‐positive	Denial of resources Depriving a woman of or monopolising her resources (bags of millet, money) Preventing women from working Asking the family of a woman who wants to separate from her husband to repay twice the dowry Prostitution of destitute women	Hitting in the face Thrashing/beating up/beating/hurting Slaughtering a daughter who refuses to marry the man chosen for her Abusing Koranic school students Kidnapping girls Beating a daughter to death because she became illegitimately pregnant	Rape (women, teenage girls and girls) Deflowering a girl Mutilating the genitals of girls Raping students

**Sources**: authors.

Table 2 provides examples of abuse that were included in both men and women's accounts. These are organised in five columns representing different types of violence based on the distinctions made by the *UN Handbook for Legislation on Violence against Women* (UN, 2010), whereby violence may be manifested psychologically, economically, physically and sexually. A fifth column was added to list all the examples of controlling behaviours that may or may not lead to other forms of violence but that restrict women and girls’ rights nonetheless.

Adolescent girls in particular are at risk of early marriage and female genital mutilation (FGM) (Amnesty International, 2011b). Despite the Prohibition of Child Marriage Act (2015), which sets the official age of marriage at 18, early marriages remain the norm in Chad. Girls continue to be the subject of family and religious arrangements as soon as they have their first period. In 2015, 30% of women aged 25–49 had married before the age of 15, and 70% before the age of 18 (EDS/MICS 2014–2015). The median age of women when they first got married was estimated at 16.1 years, whereas men tend to get married at 22.8 years.

In parallel, and despite the Reproductive Health Law (2002), which condemns all forms of violence against women, nearly two in five women (38%) in 2015 reported having been circumcised (EDS/MICS 2014–2015). However, the degree of prevalence varies considerably according to residence, ethnicity and literacy level: in Dar Sila, almost all women are circumcised (93.2%); in BeG, rates are very low (5.5%) (Alhascari and Guiryanan, 2015). In Dar Sila, a local authority representative indicated that FGM continued to be practised informally: ‘Before, circumcision was performed to a backdrop of beating drums in great ceremonies around the town. Now even those who do it, they hide in the bush in agricultural camps during the rainy season and they do it underground'.

Everyday violence, and discrimination against women and girls more generally, has multiple and cascading impacts on survivors’ ability to secure their livelihoods. The following sections look at the chain of consequences resulting from early marriage; denial of resources; abandonment of the home; and sexual violence, and their impact on the resilience capacities of survivors and those of their households.

## Impacts of violence on absorptive and adaptive capacities

### Early marriage and associated health risks

Early marriage was the most cited form of violence in interviews conducted in BeG. According to one nurse in a health centre:

*Forced marriage and child marriage are a serious health problem. This community has many cases of psychological and mental trauma, which are the consequences*.

Young people explained in a group discussion that, ‘Early marriage is a custom in our community, but a real danger for the girl: pregnancy, surgery, death'. Early marriage entails very high health risks for young girls, who are subjected to marital rape and fall pregnant before their bodies have developed to be able to give birth safely. During International Women's Rights Week in N'Djamena in March 2017, a gynaecologist at a panel discussion outlined the reproductive health implications for girls circumcised and married at a very young age:

*Girls who are married at the age of 12, they have not finished their growth, their vagina is still immature, and this type of violence sees notable complications in childbirth. These women who are victims of rape, in the home, or after a forced marriage, at a young age, experience complications during childbirth. Because they give birth at home, they do not consult a professional, and it is the traditional birth attendants who do it, and they do not take into account their size or immature pelvis; they give birth to a stillborn child and can have a fistula. These girls who suffer fistula, they are unable to control urine and stools every day, and the majority are abandoned by their husbands*.

The Centre for Reproductive Health and Fistula Repair in N'Djamena treats young patients on a daily basis: ‘We receive an average of six to eight new cases of fistula each month. In the year, we end up with 150 new cases of fistula'. The same is true in BeG, where the midwife of an urban health centre reported treating five to ten cases of early pregnancy per month.

Early marriages generate life‐threatening health risks not only for adolescent girls but also for their children. At the national level, the early age (under 20 years) of mothers represents a risk factor for infant mortality (EDS/MICS 2014–2015). This is exacerbated in rural contexts with extremely limited health services. In Dar Sila, 27% of the 47 health centres are non‐functional, and there is only one physician for 102,300 inhabitants (OCHA, 2017a). Those who need to go to the clinic usually go on foot or by donkey. However, since the law forbids early marriage, girls who need medical care are unlikely to be allowed to consult a professional. Additionally, girls and women often do not have the decision‐making power to access health services, whether for contraception, for pregnancy monitoring, for infants’ health or even for medical emergencies.

Health staff in BeG and in Dar Sila reported that villagers went to the clinic only if their husbands allowed it. According to a midwife in Dar Sila, ‘The girls here are married, and when they are sick their husbands forbid them to come to the hospital. This is violence too.’ The participants of a discussion group in Charao, Dar Sila, confirmed that:

*There are men who are really resistant to women coming to bring their children to the health centre. … They think that if the child is sick or unhappy, why should a woman come to expose this child at the hospital, it is shameful, other villagers will think that the head of household does not provide for his family, and wonder why they did not migrate to find other means*.

The influence of these social norms on men's attitudes undermines women and girls’ health and well‐being, their human capital and therefore their absorptive capacities to deal with crises. This is especially the case since they live in a context already characterised by chronic energy deficiency and malnutrition, whereby 43% and 33% in BeG and Dar Sila, respectively, are in a critical nutritional status (EDS/MICS 2014–2015). In Dar Sila, participants of a discussion explained:

*When your husband is not there, you must have the permission of his family to take a sick child to the health centre or to treat yourself. To sell the millet that is in his granary to heal yourself, his parents (his brother or father) must receive authorisation from their son before allowing it. In the meantime, you or your child could die*.

Hence, men's control over decisions and assets not only prevents women from accessing critical healthcare but also hinders collective decision‐making at the household level to support the well‐being of dependants during times of stress.

### Denial of resources, decrease in household income and increased exposure to violence

Violence has an impact on household economic capital in two ways. First, the negative impacts of violence on survivors’ health can result in extra expenditures on medical care. The law establishes that free care is available for those who are victims of violence and want to make a complaint, but in reality patients must pay (or at least advance) for medication or a medical certificate. In addition, the cost of transport is often prohibitive, as members of a group of artisans in BeG emphasised: ‘The health centre is far from us and to get there we spend 3,500 FCFA [around $6.20] to rent a round‐trip clando motorcycle. If you do not have this money you cannot heal yourself'.

Second, through limiting survivors’ physical and/or mental ability to maintain their domestic and productive activities or through imposing rigid social norms that forbid women to earn an income, violence prevents women from diversifying their resources, creating a shortfall for the entire household. This shortfall increases the vulnerability of households by limiting their resources in the event of a crisis and therefore undermining their absorptive capacities. Respondents repeatedly emphasised that husbands or relatives denied women the opportunity to engage in income‐generating activities, simply because they consider that it is not a woman's role to earn an income. This ‘denial of resources and opportunity', a form of violence cited by the majority of participants in this study, restricts female household members from accessing basic services (food, education, health, etc.). This is especially true when men do not earn a regular and/or sufficient income (particularly if they have multiple wives) and women are not allowed to start or continue running small businesses. In this case, the whole household is left without financial resources. A representative of an international NGO based in Dar Sila explained:

*You will find the husband who worked with his wife in the field until the harvest and who, after the sale, wants to get married, remarry, so he takes the whole harvest or part of it that he is going to sell to marry another woman and there, it creates problems when the woman tries to speak, either she is beaten or simply he cuts all the food for her and she ends up with nothing*.

Another representative of an international NGO based in N'Djamena emphasised these negative repercussions on the rest of the household:

*For example, the fact that the woman was beaten, she was not able to run her small business, and as a result, because she was not in the market, there were no means [money], she could not buy anything to feed the children that day. And the children go to bed hungry. And that can have several consequences. The child can end up on the street, the child can become a delinquent, he can go steal, the girl can go into prostitution, all because of the violence towards the mother*.

Testimonies in both Dar Sila and BeG also outlined that, in addition to the risk of losing their resources, women affected by disasters seem even more exposed to violence, either because situations of stress increase cases of violence or because they become subject to sexual exploitation. A representative of the Liaison and Information Cell of Women's Associations (Cellule de Liaison et d'Information des Associations Féminines, CELIAF), indicated:

*There is the reduction or even the loss of cattle because of the famine; the rains diminish and the men flee in an exodus. During such periods, there are more cases of abandonment of the home, divorces or there is an increase in the number of wealthy men who are looking to marry very young girls from poor backgrounds. The dowry is not very significant: on average one cow or two goats, plus 100,000 FCFA [about $178]*.

Several interviews conducted in BeG highlighted that household poverty was a risk factor driving early marriage. A representative of an international NGO believed that, ‘Often it is the influence of the rich on the poor. Rich parents seduce poor parents who then put pressure on their daughters'. Amnesty International (2011a) also reports increased vulnerability of adolescents where parents lack the means to provide for the family. In these cases, women may be sexually exploited for protection or food. This form of violence implies forced/unwanted sex in exchange for material resources, services or assistance (UNHCR, 2003).

Sexual exploitation thus becomes a survival strategy in times of crisis, allowing women and girls to maintain the minimum of their economic resources while the dowry provides some families with a source of punctual income. The mobilisation of such survival strategies, which in itself constitutes a form of VAWG, is a paradox that echoes the literature on risks and disasters: to access livelihoods that allow for survival in the short term, strategies that are being mobilised may increase the vulnerability of those most at risk in the long term (Wisner et al., 2012). This vulnerability to pre‐ and post‐crisis violence thus doubly limits survivors’ capacities to cope with shocks and stresses.

### Male migration and pressure on female‐headed households

In a context of chronic food shortage, widespread domestic violence and economic fragility, it is difficult to separate the risks associated with climate change from ‘everyday’ risks and, therefore, to differentiate between coping strategies and adaptation strategies (see also Wisner, 2018). In other words, each strategy is supposed to protect and develop people's human resources (to heal, to educate, to provide training), increase productivity, diversify incomes or enable voices to be heard in community decisions.

Temporary migration is one such strategy, mostly developed by men, since women are restricted, and even denied their freedom, in terms of migrating and engaging in productive activities, owing to gender norms around men and women's assigned roles in the household. Men thus seek new sources of income in other parts of Chad, or abroad, in Libya or Sudan. Male farmers in a group in BeG explained that, ‘In the past 20 years, because of climate change, men have started to move away because rain‐fed crops are no longer producing anything. It was at this moment that women took over from men in agriculture'.

When this migration allows men to access new sources of income, improve their conditions, share their wealth and provide for their household, it becomes a widely mobilised adaptation strategy. However, the majority of people interviewed in Dar Sila felt that men's mobility negatively affected the family, because men do not necessarily bring back new resources, or they simply never come back, because they have remarried and settled elsewhere. Even if they aim to come back eventually, the majority do not have the means to transfer the money earned in a context where financial transfer systems are very poorly developed. Only 15% of households reported having received cash transfers in 2015, a slightly higher percentage than in 2012 (11%) (Concern Worldwide and Feinstein International Center, 2016a, 2016b). Villagers from Dar Sila illustrated this point:

*The big problem in our community is the departure of men. They go in search of gold and this can last two years or more. For example, I was three months pregnant with this child I am holding now when his father left and I have no news. All these men who go away saying they are looking for gold, we, women, have not seen anything yet. They come back empty handed and will still help themselves to our savings*.

Asked whether natural phenomena, such as droughts or floods, or even conflict, influenced inequalities, a local authority representative in Goz Beida explained:

*It accentuates inequalities because people leave, they go to Libya, they go to Sudan, or within the country, or in the quarries to look for gold. But these people, most often, leave the workload to the families, and they bring back nothing. Even those who find something, they settle there, it is only when there is a surplus that they think of the family*.

The capacities of household members who stay behind are affected all the more negatively if the man who has migrated does not allow his wife to develop productive activities in his absence, and women are supposed to wait for the return of their husband. Many women do not know if and when their husband will return but they cannot remarry or control the (ex‐)husband's resources, such as the land or the granary, to feed their children, which further exposes them to exploitation and violence.

**Figure 1:**
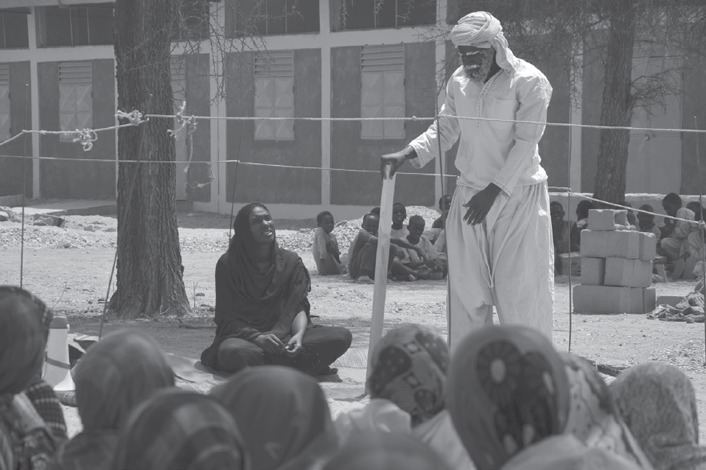
Play performed in front of the villagers of Ngorloli, Dar Sila, organised by Concern **Source**: Virginie Le Masson, March 2017.

To help tackle this issue, a play was presented in the village of Ngorloli on the occasion of International Women's Rights Week in March 2017, as an initiative of Concern's BRACED project in Dar Sila. The play depicted the difficulties facing the wife and daughter of a man who went to search for gold because he forbade them from continuing trading or going to school in accordance with their gender and associated roles. To survive, the mother decided to ignore the ban and sell vegetables in the market, which also allowed her to pay for the schooling of her daughter. The husband returned years later, without any income (see Figure 1).

Respondents in Dar Sila and BeG also pointed to migration of men as a strategy to allow men to build economic resources in order to marry new women, whether in their village of origin or elsewhere. Villagers in a group discussion in Dar Sila said that men grabbed and kept resources, deserted the household and took new wives. Mothers or daughters who remain need to earn an income to support themselves, which increases their exposure to the risk of violence through sexual exploitation; to the risk of having to give up school and/or of forced marriage, to provide the family with an additional source of income or dowry; and to the risk of verbal, physical or sexual assault by other members of the household or village, in the absence of a (protective) husband or father. The head of a health centre in BeG explained:

*These cases of violence are more and more recurrent in the past five years and especially during seasonal food shortages, when the husband must go out of his way to provide for his family. Sexual violence and fighting in the home is especially common. All communities are affected. Women are more exposed because of the absence of the husband. This violence has a detrimental effect on their ability to cope with everyday life*.

These testimonies differ from those of some local authorities, such as a village chief in Dar Sila, who felt that women ‘stay in the village with the children and must wait for their husband because he is gone for their well‐being. A good woman must endure this absence'. This view echoes a report by the Chadian government (MSP, 2014) that underlines the low status of women in the productive economy, often in food and livestock activities, and the way they are limited in terms of applying strategies to adapt to climate changes. At a community level, men's migration also entails a trade‐off between the potential benefits for their household of the resources they seek and the cost of their absence for the proper functioning of the village institutions. Concern's reporting from its BRACED project indicates that water committees, for example, do not operate all year round because some members migrate during the dry season (Concern Worldwide, 2017). Such consequences affect negatively the adaptive capacities of households and communities because imbalanced decision‐making processes undermine intentional and incremental adjustments to be made in anticipation or response to stresses (Jeans et al., 2017).

### Sexual violence, lower social resources and restricted education

Attitudes and behaviours related to the domination of women and girls by household, family or community members govern social norms that legitimise polygamy, do not tolerate extramarital pregnancy and hold girls and women responsible if they become pregnant outside marriage. The consequences for not abiding by these norms are highly detrimental for the social resources of girls and women. According to a midwife at a health centre in BeG, a girl who has experienced rape and/or pregnancy outside marriage may suffer the same fate: ‘No one else is going to marry you; your parents will throw you out'. The president of a national women's rights association in BeG said girls and women saw negative impacts on their social resources even in the case of consensual sex:

*Sex before marriage takes place in the school environment. But the girl who falls pregnant is rejected by her family and in‐laws. The child is declared illegitimate. There have been cases where the girl leaves the house to flee with the father [of her child]. Parents are often very angry when it happens to their daughter. There was a case where the father beat his daughter to death*.

Even when girls have been subjected to sexual violence, they are often held responsible by both men and women family members, for the occurrence of violence and the potential resulting pregnancy. A canton chief in Dar Sila highlighted the situation for women who are alone at home: ‘If the woman is confident, she refuses [the advances]. The non‐confident let it happen, that's where there are problems'. A representative of an association that advocates for women's rights also reported:

*There was a case of rape of a 14‐year‐old girl by a soldier. She became pregnant and gave birth to a girl. Her father said he didn't want to see the baby in his home. The girl ended up throwing her baby in the trash. In the morning, the baby was found alive and after an investigation the girl was found. Considered guilty, she ended up being sentenced to three years in prison in N'Djamena*.

Hence, women and girls are not only more exposed to sexual violence but also suffer more from the consequences of discriminatory gender norms, including rejection by their family and community. This further undermines their social resources and overall livelihoods. A young mother in BeG told us what happened after she fell pregnant out of wedlock and the child's father died in an accident:

*When this pregnancy was revealed, my parents disowned me and asked me to leave the family home. I went to a friend … and I left school before giving birth because it was unbearable as I did not have any support during my studies. When the others have school equipment and clothes and you have no one to support you it is difficult to live. My family says that I brought shame upon them and that I am disgraced. I will not do that to my daughter if she happens to be in this situation one day … [tears]*.

The linkages between girls’ exposure to sexual violence, and to male dominance more broadly, and their restricted access to school is particularly detrimental for their capacity to develop their education. In Dar Sila and BeG, respectively, 90% and 92% of women have never been to school, compared with 70% and 82% of men (EDS/MICS 2014–2015). The director of a college in BeG reported that girls represented only 26.8% of his students, and that these mostly came from a so‐called higher caste. This context limits the opportunities for girls and women to know their rights and to access information on their reproductive health.

Lack of reproductive health and sex education within families perpetuates the tendency for girls to be blamed in cases of sexual assault and unwanted pregnancy and limits the access of young people, but also of parents, to health services for contraception or protection. According to the EDS/MICS 2014–2015, only 15% of women (against 57% of men) think it is normal for a woman to refuse to have sex with her husband or partner if she knows he is having sex with other women. In addition, only 14% of women (70% of men) think it is justified for a woman who knows her husband has a sexually transmitted disease to use a condom during sex. Such disparities between men and women's views could be explained by conservative gender norms in Chad that maintain women in a subordinate position, with low access to information and lack of power to decide over their integrity. In contrast, the proportion of women favouring these two protection measures increases with their level of education and is higher in urban than in rural areas.

Lack of access to schooling for girls and women and the resultant illiteracy also have an impact on family health, particularly children's vaccination coverage. For example, the EDS/MICS 2014–2015 shows that fewer than one in five children (19%) receive all recommended vaccines when the mother has no education, compared with 33% when she has attended primary school and 46% when she has had a higher level of education. The sexual, physical and psychological violence that affects girls, restricts their social capital and limits their ability to attend school and access education maintains the long‐term vulnerability of survivors and that of their household by restricting future opportunities for girls to access more lucrative income‐generating activities, to develop their knowledge and to contribute to decision‐making.

## Impacts of violence on transformative capacities

The goal of transformation is to eliminate or reduce risk factors, vulnerability and inequality, in order to lessen the long‐term impact of crises on the poor and the victims of discrimination as a priority. Transformation differs from adaptation in that it deliberately seeks to change the state of being, instead of (simply) adjusting practices to fit new conditions (Few et al., 2017). It is therefore a long‐term process that requires sustained social changes through transforming norms, attitudes, practices and beliefs that currently tolerate VAWG.

First, social change is limited when survivors do not have decision‐making power. However, participation in decision‐making at the household level can also be a risk factor for violence when or where unbalanced and abusive gender power relations exist. The proportion of women who have experienced spousal violence is in fact lower (29%) among those who have not been involved in decision‐making processes than it is for those who have (36%) (EDS/MICS 2014–2015). According to a report by the gender advisor of an international NGO, domestic violence discourages women from engaging in negotiations with their husbands on topics they know are sensitive. The women the advisor had interviewed in communities in Dar Sila had said it would be unimaginable for them to ask their husbands for money to buy clothes or underwear, as this request would undoubtedly lead to an argument and often to violence.

It is also difficult for women who have been abandoned by their husbands to conceive of the possibility of remarrying, or to question polygamy. A woman participant of a focus group discussion in Dar Sila explained that, ‘If you get angry and leave home, it's to put the lives of your children at risk. The man will marry another woman and turn his back on your children'. Overall, many interviews revealed the lack of general consideration of girls as right‐holders and the lack of respect for them as agents of their own development. According to a canton chief:

*If the girl is old enough to marry [when she has had her period] then she must obey. … The man is superior to the woman. The woman is below him. There is no equality because the woman depends on the man. And that will not change*.

Second, limited access to formal education constrains people, and women in particular, from accessing decision spaces. Illiteracy affects more than two out of three people in Chad (UNICEF, 2010), while only 40% of over‐15s could read and write French or Arabic (48% of men compared with only 32% of women) in 2015 (UNESCO, 2015). This means that people's knowledge of the legislative framework that officially condemns discrimination and violence on the basis of gender is limited. It also prevents women from claiming their rights and constrains their equitable representation in politics. The National Assembly has only 24 women deputies, out of 188 (less than 13%) (IPU, 2017), which may explain in part why issues of equality and women's rights are not treated as a priority. The lack of access to information and communication channels maintains the social exclusion of women and girls from the public sphere and the abuse of power, and thus limits transformation.

Third, transformation is compromised by the lack of access to justice. The governance system is characterised by the coexistence of civil law, customary law and Islamic law. The multiple justice systems and the confusion as to the status of women in the family limit the application of the principle of equality affirmed in national legislation and international texts ratified by Chad. In customary law, marriage, property and succession remain determined by sex and masculinity is prioritised (MSP, 2014). As a result, women's decision‐making power over family planning is restricted (such as on birth spacing or limitation) and they do not exercise the same responsibilities as men on an equal basis, during marriage or when it is dissolved (MSP, 2014).

In eastern Chad, formal justice institutions are also very limited or non‐existent, and rural residents depend on customary courts presided over by traditional leaders (IDMC and NRC, 2010). Complainants often prefer these courts because of the higher speed of judgement, and because of the uncertainty and difficulty involved in engaging in a formal, non‐functioning judicial system (IDMC and NRC, 2010). The Association for the Promotion of Fundamental Freedom in Chad (Association pour la Promotion des Libertés Fondamentales au Tchad, APLFT) provides free legal advice to those who are aware of this service and have the means to go to urban centres to talk about their case. In 2016, 10 survivors asked APFLT in Dar Sila for legal advice and assistance in drafting their complaint in French, filing it with the police and drawing up a report. However, social norms value the authority of customary institutions to settle conflicts as privately as possible. A police officer explained that, ‘For a man, it's not good to leave his wife to the police. … It is recommended that business be settled at the chief level.’ Typically, women have barely any access to governmental authorities other than the village chief, the imam, the canton chief, the judicial police officer and the sultan (in Dar Sila) – the majority of whom are men. A village chief explained the standard for responding to violence:

*Normally, we prefer to settle domestic violence [issues] at home. It's not good for a woman to expose her husband. We go to the police if there is blood, theft, adultery. For example, we just had a case where the husband broke his wife's tooth. At the level of the police, the husband has paid a sum for damage to the victim but we have closed this problem between us here because it will take a lot of time if we go to Goz Beida for justice and it will also destroy social cohesion*.

In rural areas, the proximity between community members and traditional chiefs as dispute mediators could encourage accountability among leaders because their decisions and impacts come under direct observation. However, the widespread impunity of perpetrators of violence reflects unsatisfactory application of the law and a lack of capacity of women and their families to hold leaders responsible for ensuring their protection. This is reflected in the discourse of some authority representatives, such as this police officer:

*Before there were forced marriages, yes, but since the law came into effect and the increased awareness, this has ended, the cases of violence, all that, everything is back to normal, it's over, it is the girl who now chooses her husband, and this change is not just in cities. If someone says there is a forced marriage, he's a liar*.

If authorities themselves deny, hide and tolerate violence, this considerably limits survivors’ opportunities to complain, access medical, judicial and psychosocial support services and hold leaders accountable for crimes or for making decisions that run counter to human rights and sustainable community development. An NGO representative in Dar Sila flagged the issue of *'culturally acceptable violence*', whereby, ‘*Men are facilitated by certain cultural laws and are not held accountable.'* A local authority representative in Dar Sila also indicated that official complaints were rare:

*They [the survivors] find it a shame [to complain], they think they are exposing the husband. Because even if they come here, when we cannot find a compromise, and we try to get the husband to the police, they think, ‘No, we'll [the police] ransom him, we'll pull out the little bit they have, we will make him suffer.’ And after the community will reject her. So … the law they apply is often silence*.

A gynaecologist at the N'Djamena Centre for Reproductive Health and Fistula Repair confirmed that:

*A raped woman is ashamed, she is stigmatised. She does not automatically come to the hospital. … The majority do not report abuse and therefore we do not know the exact number of raped women or children. … Parents hide children at home. So when there is a case, when it's the neighbour [the perpetrator], when it's the uncle, when it's someone close, we're afraid to reveal because he can end up in prison and it can become a conflict, a family drama*.

Lack of access to protection and justice results in a culture of silence, which in turn does not contribute to social change but rather maintains discriminatory norms that endanger the health, dignity, safety and autonomy of survivors and undermine their resilience. At the national level, it is estimated that almost half of all women who have experienced psychological, physical or sexual violence do not seek help or tell anyone what had happened to them (EDS/MICS 2014–2015). The weight of social norms that discriminate against women and girls and the resulting violence exacerbate power inequalities and limit survivors’ ability to challenge the established order.

## How can resilience programmes address violence against women and girls?

This analysis of how VAWG reduces resilience capacities illustrates the role social justice must play in building resilience (Sotelo, 2017). However, development actors must also implement more transformative approaches, for example by denouncing discrimination against women and girls. Development programmes that aim to build resilience need at a minimum to avoid perpetuating gender inequalities, and can support several processes of social change to tackle violence and build resilience at individual, household and community levels.

**Avoid perpetuating gender‐based inequalities**: Research findings show that norms that discriminate against women and girls limit their participation in decision‐making bodies, in local development structures, in the workplace and even within development programmes. For example, very few positions in NGOs in Chad are accessible for women, and practitioners reported having difficulties recruiting women. Not only are the education levels and degrees sought in the desired profiles out of reach for many women in regions such as Dar Sila or BeG, but also certain practical requirements (e.g. ability to drive, travel, attend training for several days), as well as gender‐based discriminatory attitudes and power imbalances that exist within organisations meant to tackle them, constitute employment barriers. According to a Chadian representative of an international NGO in N'Djamena:

*The biggest challenge is the understanding of gender by the staff and the communities that we serve. Most of the time questions asked by women and young girls are relayed to the lowest position. … The place that organisations provide for women is a real challenge. … Women's views are not heard because of their limited numbers. They cannot pass information on regarding gender concerns, even within their own organisation*.

An explicit equity policy can help in redesigning recruitment processes, setting up awareness‐raising for staff and identifying project goals on eliminating gender‐based discrimination and supporting equality. Otherwise, the design of projects may be affected by lack of information on women's roles and opinions in communities affected by crisis, lack of gender and power analysis and lack of insights into the risks facing the most marginalised community members and the possible protection mechanisms that could be supported to help resilience.

**Help maintain protection mechanisms implemented by humanitarian responses**: Clinical management of GBV cases in general, and rape in particular, according to the WHO standard protocol, remains a major problem in Chad and in BeG specifically. At one health centre in BeG, staff explained that health care with regard to rape is provided only for traumatic injuries and the treatment of sexually transmitted diseases. In addition, no support is offered to access justice, while the shame many survivors and their families feel, combined with limited income and a lack of trust in institutions, discourage and prevent them from using available services when they exist.

In Dar Sila, the risks of GBV are in fact better addressed in the humanitarian response than in development programmes, thanks to the coordination by the UN Protection Cluster of relevant programmes. Interviews with NGOs, UN agencies and state protection parties revealed that, during emergency interventions, a referral system is set up to coordinate the provision of health care to survivors of violence and establish prevention activities. In contexts of insecurity, organisations typically see an increase in the number of cases of violence, not only because the crisis exacerbates vulnerabilities but also because the existence of a system of protection encourages survivors to report cases. As indicated by representative of a UN agency in N'Djamena:

*In villages, violence is considered normal for women, trivialised. So it is with the emergence of humanitarian parties who work in the context of prevention of GBV … they arrive, will raise awareness with the population, they make services available, they manage to gain the population's trust, this is where we know cases of GBV*.

This protection system is still functioning in internally displaced person camps in Dar Sila, with well‐defined responsibilities distributed between the actors involved and a database of recorded cases of abuse, which is useful for any initial risks assessments to better understand the problems facing local populations and the factors that prevent them from coping with crises. However, the humanitarian protection system does not support members of local communities (only displaced persons and refugees) and it ends with the departure of humanitarian actors. The conflicts in eastern Chad may have ceased, but chronic food shortages prevail. This means a permanent environment of emergency is in place, in which women and girls remain the main victims of sexual exploitation, domestic violence and abuse of power. The lack of sustainability and integration between humanitarian responses and development initiatives to prevent and respond to violence means that the risks of suffering from, and perpetrating, violence remain largely unaddressed.

**Support girls and women's reproductive health rights**: Civil society initiatives to provide information on women's rights and family planning do exist – for example the Chadian Association for Family Well‐Being (Association Tchadienne pour le Bien‐Ětre Familial, ASTBEF), CELIAF and many more. One UN report (UNFPA, 2016) points to the relative success of these initiatives, including ASTBEF youth reproductive health clinics in N'Djamena, which provide adolescents with access to spaces that meet their expectations and needs. However, better access to reproductive health services, especially in remote and rural areas, is still needed.

**Collaborate with women's groups**: Women belonging to a community group emphasised the benefits they derived from developing their livelihoods and accessing information. According to the president of CELIAF in BeG, ‘The women who form groups are better able to defend their interests.’ Many NGOs thus support village associations, especially women's groups, where members pay a monthly contribution and benefit from grant support credits and access to information. In Dar Sila, community action committees, with an average female membership of 44.2%, rely on newsletters and the risk analyses shared by Concern and the Sustainable Information System on Food Security and Early Warning to set up small activities to reduce the impact of shocks (Concern Worldwide, 2017). To respond to the risk of crop failure, two of these groups built community granaries to store sacks of millet and/or sorghum for use in emergencies (Concern Worldwide, 2017). In BeG, the priority of the regional government representative for social affairs is to create training centres, businesses and jobs for women and to give them access to credit. Investing in women's groups and supporting their income‐generating activities is also a way to transform power hierarchies, an approach many organisations follow in the belief that economic empowerment will enable women to emancipate themselves.

**Work with authorities, including traditional and religious leaders**: Participants in this study pointed out the need to invest in raising the awareness of authorities, especially traditional leaders, to facilitate the dissemination of laws and involve them in changing behaviour, given their influence in communities. This begins with sensitising religious and traditional leaders on the risks associated with harmful practices such as FGM and the physical and psychological impacts of violence, as well as on what the law says. Better informed by medical practitioners, psychologists, magistrates or theological specialists who clarify what is stated in holy books in terms of rights, traditional leaders can get involved in advocacy efforts against violence in their community and other villages. UN agencies and NGOs are developing such strategies to tackle VAWG while respecting the social hierarchy, so as to avoid a hostile reaction at the beginning of projects and maximise the effectiveness of outreach activities by obtaining support from those who control power. Relying on government representatives to share information on the rights of citizens and spread messages is also an effective strategy to change attitudes toward inequalities, because information is conveyed by a man, in the local language and based on the law and on religious texts.

**Create large‐scale awareness of violence against women and girls**: There is an urgent need to inform women and girls of the laws that protect them. One of the main aims of the Association des Femmes Juristes du Tchad (Association of Female Lawyers of Chad, AFJT) is to allow women to learn about their rights as stipulated in the Constitution and the laws of the Chadian government. Radio programmes are often cited as a means of increasing access to information and awareness on a large range of issues related to law and equality. NGO staff members also play a critical role in informing populations of their rights, even if they work in different sectors than protection. Hence, they need to have assessed the risks facing community members, especially the most marginalised, particularly if projects are intended to promote the change of discriminatory behaviours.

Therefore, effective prevention of violence requires government and NGOs to increase the number of initiatives in different sectors, through the largest possible number of development actors, because a single programme cannot undertake mass awareness‐raising. Good practices that make people aware of their rights, such as dialogues between local authorities and the population on equality during Women's Day celebrations, are examples to support. Practitioners also highlighted initiatives that include and rely on men and boys as crucial to change discriminatory attitudes.

**Contribute to address VAWG and respond to survivors’ needs through quality holistic service provision**: Informing women of their rights and sensitising local populations on the problem of violence, where it is considered the norm, raises the risk that VAWG will increase, because traditional power relationships are being threatened (McDermott and Garofalo, 2004). Thus, in addition to sensitisation, a system of response to violence must be put in place locally. This requires state involvement, as such a system must rely on the national legislative framework and functioning judicial institutions (Morrison, Ellsberg, and Bott, 2007). NGOs can contribute by referring survivors to health centres, hospitals and paralegal associations, but, without an adequate legal system, survivors will continue to be discouraged from seeking support. Supporting the resilience of survivors of violence entails setting up a social protection system in regional centres, integrating a process of psychosocial support into health centres and establishing a community and family support system specific to Chad's culture and the specificities of each region of the country. The work being done by CELIAF goes in this direction, and initiatives can build on the work done by humanitarian actors.

**Mainstream gender equality in cross‐sectoral resilience programmes**: Since gender inequality is the norm, resilience programming that aims to transform norms needs to address gender issues in a progressive way, alongside other activities in different sectors, as suggested by the representative of an international NGO based in Dar Sila:

*They [local authorities] see people who arrive to disturb their culture, their habits, because women have never complained before so why is it that others should come forward to tell them, no, that's not good, whereas before their society worked. Before when a man was allowed to hit his wife, it was good. When they prevented girls from going to school, that was fine … it was the norm. Then you arrive with your documents, your papers and your human rights, you tell them that, it's not good. … It is not easy for the local authorities to accept this, to recognise your concerns or for them to accept external help in this area. If they want you to help with drilling a bore hole, of course, they'll call you, if they want to make a road or a bridge, build a school, they'll call you. Now if you were to approach the issue of gender in a cross‐disciplinary way then that would be fine*.

A cross‐cutting gender approach, advocated by many participants in this study, echoes the cross‐sectoral protection recommended by humanitarian advocates who wish to incorporate protection principles into programmes as a means to promote meaningful access to humanitarian assistance, and to advance the safety, dignity and respect of those in affected communities (Slim and Barwick, 2005; OCHA, 2016). Such an approach makes it possible to address people's basic needs while progressively addressing the constraints rooted in social norms. The goal is to foster processes of social change in the long term, through changes in behaviours, perceptions and socio‐cultural norms.

At the same time, however, these changes must be accompanied by other, shorter‐term, measures that respond to the immediate needs of survivors, their families and their communities. In an economic context as fragile as that of Chad, basic needs are enormous – access to water, food security, health, hygiene and sanitation, land, markets, transport and communication infrastructure, etc. These are the many needs that make NGOs’ activities indispensable when investments and social protection from the state are lacking, and that make humanitarian and development intervention useful to local populations. Staff at a health centre in Dar Sila confirmed that a cross‐cutting gender approach would be welcome in activities that support agriculture or sanitation, but that it must be ‘implicit':

*You work on what they want first, and then you can propose what they do not want to do∼ But it is necessary to conduct a pre‐investigation, and to target the imams … and to ensure that the women [the community focal points] are well trained and can attend to these women [the survivors] but crucially that they do not expose them or do anything to destroy the couples. NGOs should not visibly display violence … it must be done in a different way … like theatre plays, so they recognise their faults*.

A cross‐cutting gender approach can help change the way people live without threatening cultural identity, through projects on health, nutrition and agriculture that convey information and messages on men, women and children's well‐being. For example, when implementing activities to tackle malnutrition, doctors and nurses can explain to fathers and mothers that children are more likely to face health problems when the mother is very young. Programmes elsewhere in Chad have relied on the involvement of imams, who integrate messages on pre‐natal health during prayer gatherings targeted at men so they allow their wives to go the health centre. Showing examples of what works well elsewhere and the benefits that have been generated, not only for women but also for everyone in the household and for the whole community, is also an awareness tool: a man whose wife earns an income that benefits the entire household may be more willing to recognise her rights.

## Conclusion

The many types of VAWG constitute a manifestation of unequal power relationships that have negative impacts on the livelihoods of survivors and of their families. This paper has examined how everyday violence affects the resilience capacities of survivors and people affected by crises. Based on analysis of secondary data at national scale and on the accounts of development practitioners, authority representatives and the inhabitants of two regions of Chad, Dar Sila and BeG, it shows that violence negatively affects resilience‐building because it prevents survivors, and their dependants, from proactively and positively managing ongoing challenges and crises. Gender inequalities, and even more so violence against women, lead to a chain of problematic consequences, from precarious reproductive health to a low literacy rate, which primarily affect women. The resulting limited livelihoods constitute a risk factor for VAWG, especially sexual exploitation, by increasing women and girls’ vulnerability. In summary, violence affects the livelihoods of survivors while their lack of resources increases the risk of violence.

A number of (often informal) institutional obstacles also prevent women from accessing the same opportunities as men, including norms that limit women's mobility, their control over financial resources and their access to justice, and overall pervasive gender imbalances across all life domains. These obstacles also stop women exercising decision‐making powers on an equal footing with men, which limits the resources women are able to mobilise in the event of a crisis, therefore undermining their absorptive and adaptive capacities. If resilience is ‘the ability of women and men to exercise their rights and improve their well‐being despite traumatic events, stresses and uncertainty’ (Jeans et al., 2016), then VAWG undermines the capacities of survivors to cope with crises.

A transformative approach is needed to address the structural causes underlying discriminatory inequalities and gender norms. Development programming can establish interventions that: concretely, albeit indirectly, challenge existing imbalances; provide useful services to women, survivors and community members; and increase the visibility of the problem of violence, through the documentation of abuse and the denial of rights and through multi‐sectoral collaboration. In this way, it can contribute to the transformation of unequal power relationships to help build resilience in places that are exposed to natural hazards and climate change.

## Acronyms

AFJT: Association des Femmes Juristes du Tchad (Association of Female Lawyers of Chad)
APA: Agence de Presse Africaine
APLFT: Association pour la Promotion des Libertés Fondamentales au Tchad (Association for the Promotion of Fundamental Freedom in Chad)
ASTBEF: Association Tchadienne pour le Bien‐Ětre Familial
BeG: Bahr‐el‐Gazal
BRACED: Building Resilience and Adaptation to Climate Extremes and Disasters
CELIAF: Cellule de Liaison et d'Information des Associations Féminines (Liaison and Information Cell of Women's Associations)
DFID: UK Department for International Development
EDS: Enquěte Démographique et de Santé (Demographic and Health Survey)
FGM: female genital mutilation
GBV: gender‐based violence
HIV: Human Immunodeficiency Virus
IASC: Inter‐Agency Standing Committee
IDMC: Internal Displacement Monitoring Centre
IFRC: International Federation of Red Cross and Red Crescent Societies
INSEED: Institut National de la Statistique, des Etudes Economiques et Démographiques
IPU: Inter‐Parliamentary Union
MICS: Multiple Indicator Cluster Survey
MSP: Ministère de la Santé Publique, de l'Action Sociale et de la Solidarité Nationale
NGO: non‐governmental organisation
NRC: Norwegian Refugee Council
OCHA: UN Office for the Coordination of Humanitarian Affairs
RFI: Radio France Internationale
UK: United Kingdom
UN: United Nations
UNESCO: United Nations Educational, Scientific and Cultural Organization
UNFPA: United Nations Population Fund
UNHCR: United Nations Refugee Agency
UNICEF: United Nations Children's Fund
VAWG: violence against women and girls
WHO: World Health Organization

## Acknowledgements

The authors would like to thank all of the respondents to this study as well as the reviewers for their positive and encouraging comments.
